# Cell–Cell Adhesion and Myosin Activity Regulate Cortical Actin Assembly in Mammary Gland Epithelium on Concaved Surface

**DOI:** 10.3390/cells8080813

**Published:** 2019-08-02

**Authors:** Wei-Hung Jung, Khalid Elawad, Sung Hoon Kang, Yun Chen

**Affiliations:** 1 Department of Mechanical Engineering, Johns Hopkins University, Baltimore, MD 21218, USA; 2 The Institute for NanoBioTechnology, Johns Hopkins University, Baltimore, MD 21218, USA; 3 Center for Cell Dynamics, Johns Hopkins University, Baltimore, MD 21205, USA; 4 Department of Materials Science and Engineering, Johns Hopkins University, Baltimore, MD 21218, USA

**Keywords:** curvature sensing, adherens junction, E-cadherin, cortical actin, myosin

## Abstract

It has been demonstrated that geometry can affect cell behaviors. Though curvature-sensitive proteins at the nanoscale are studied, it is unclear how cells sense curvature at the cellular and multicellular levels. To characterize and determine the mechanisms of curvature-dependent cell behaviors, we grow cells on open channels of the 60-µm radius. We found that cortical F-actin is 1.2-fold more enriched in epithelial cells grown on the curved surface compared to the flat control. We observed that myosin activity is required to promote cortical F-actin formation. Furthermore, cell–cell contact was shown to be indispensable for curvature-dependent cortical actin assembly. Our results indicate that the actomyosin network coupled with adherens junctions is involved in curvature-sensing at the multi-cellular level.

## 1. Introduction

It has been shown that cells respond to the geometry provided by their microenvironment. For example, the barrier functions of the intestinal epithelial cells are modulated by the local villi curvature [1]; tissue geometry dictates breast cancer cell tumorigenicity [2] and invasiveness [3]. The effect of substrate curvature has also been reported as a mechanical cue affecting single-cell migration [4,5] or collective migration [6,7]. Though several membrane-associated proteins have been identified in responding to curvatures at the sub-micron scales [8,9,10], it is not clear how cells sense curvatures at the multicellular level.

To identify the underlying mechanisms for curvature sensing at the scale of tens of microns, 3D-printed channels were deployed to grow epithelium at the curvature of our interested scale. This model system is in mimicry of the geometry observed in the glandular ducts in vivo, commonly seen in the lung, kidney, prostate, liver, circulatory system, and mammary gland [11]. Cell migration and cell-substrate adhesion were reported to be affected by the curvature at the scale of tens of microns [12,13,14,15], suggesting possible involvement of actin cytoskeleton and adhesion molecules present at the cell surface. Therefore, in this study, we evaluated the actin distribution and adhesion protein expression in cells on the curved surface.

We chose to study the phenotypical differences of mammary epithelium on the flat surface and curved channels because the results might be indicative of how breast cancer cells progress and metastasize differently on varied curvatures. There have been recent studies where epithelial cells exhibited different migration patterns when passing through cylinders of different diameters [6], or when moving to close the gaps with varied curvatures at the x-y plane [7]. Our model system allowed us, on the other hand, to study the stationary epithelium on the curved surface. We observed increased cortical actin formation and E-cadherin expression in cells growing on the curved surface with a curvature of 1/60 µm. Drug inhibition experiments showed that myosin II activity contributes to the elevated cortical actin assembly on the curved surface. Our study confirms that curvature sensing at the scale of tens of microns can be achieved through the actin cytoskeleton-adherens network which connects single cells in the epithelium.

## 2. Materials and Methods

### 2.1. Cell Culture

EpH4-EV cells were used in all experiments and cultured in DMEM high glucose medium (Thermo Fisher, Waltham, MA, USA), supplemented with 10% FBS (Thermo Fisher) and 1% penicillin-streptomycin (Thermo Fisher, Waltham, MA, USA) at 37 °C and 5% CO_2_. 5 × 10^5^ cells were seeded on the PDMS substrate with a total area of 300 mm^2^, where curved open channels were intervaled by flat regions, and cultured for 1 day. The cells on the flat region of the PDMS substrate were used as control. For the low-density experiments, 10^4^ cells were seeded on the substrate with a total area of 300 mm^2^ and cultured for 1 day.

### 2.2. Image Acquisition

The images of cells were acquired using a Leica SP8 confocal microscope with 40X water immersion or 63X oil immersion objectives. Z stacks with the pinhole set at 2 airy units and the step size of 0.76 μm were obtained to cover from top to survey the cells distributing across the curved structure. To quantitatively compare fluorescence intensity, for each specific staining using fluorophore-labeled chemicals or antibodies, images of cells in different groups were acquired using the same laser power, the same pixel dwell time and same gain values for the photomultiplier.

### 2.3. Drug Treatment

5 × 10^5^ cells were seeded on the PDMS substrate with a total area of 300 mm^2^ for 1 day, followed by the treatment of Y-27632 (Cell Signaling Technology, MA, USA, 13624) at 10 μM for 2 h or of Blebbistatin (Toronto Research Chemicals, North York, ON, Canada, B592500) at 25 μM for 2 h, to inhibit Rho activity and Myosin II activity, respectively. For beta-catenin inhibition, 5 × 10^5^ cells were seeded on the PDMS substrate with a total area of 300 mm^2^ and cultured for 1 day, followed by treatment of XAV-939 at 10 μM (Selleckchem, Houston, TX, USA S1180) for 24 h.

### 2.4. Immunofluorescence and Staining of F-Actin and the Nucleus

Cells were fixed in 4% paraformaldehyde for 10 min and then washed with PBS. The cells were then permeabilized in PBS with 0.5% Triton X-100 (Sigma-Aldrich, St. Louis, MO, USA, X100) for 10 min. After washing, the fixed cells were blocked to prevent non-specific binding using PBS containing 5% normal goat serum (Sigma-Aldrich, G9023) for 30 min. For the staining of E-cadherin, rat anti-E-cadherin antibody (Abcam, Cambridge, MA, USA, ab11512) was diluted 1000 times, and its corresponding secondary antibody, goat anti-rat IgG antibody conjugated with Alexa 488 (Jackson ImmunoResearch, West Grove, PA, USA), was diluted 500 times. For the staining of pMLC, rabbit anti-Myosin light chain (phospho S20) antibody (Abcam, ab2480) was diluted 1000 times, and its corresponding secondary antibody, goat anti-rabbit IgG antibody conjugated with Alexa 594 (Jackson ImmunoResearch), was diluted 500 times. For the staining of total MLC, mouse anti-Myosin antibody (Abcam, ab24648) was diluted 250 times, and its corresponding secondary antibody, goat anti-mouse IgG antibody conjugated with Alexa 647 (BioLegend, San Diego, CA, USA, 405322), was diluted 250 times. All the antibody was diluted in PBS with 2.5% normal goat serum. The fixed cells were incubated with the primary antibody for 2 h at room temperature, followed by washing using PBS three times and then incubating with the secondary antibody for 1 h. For staining of the F-actin and the nuclei, actin-stain 670 phalloidin (1:500; Cytoskeleton Inc, Denver, CO, USA, PHDN1) and DAPI (1 μg/mL; Sigma-Aldrich D9542) were used respectively for 30 min of incubation. After staining, the PDMS substrate was then mounted cell-side down on the cover glass with two drops of ProLong glass antifade mountant (Thermo Fisher Scientific, P36982) for mounting. The mounted samples were left in room temperature for 2 h to be dried and then stored at 4 °C for imaging.

### 2.5. Concaved Channel Fabrication

#### 2.5.1. Negative Mold Fabrication

The negative molds were printed using the Asiga PICO2 3D printer. The printed molds were briefly washed in Isopropyl alcohol and then further crosslinked in Asiga Flash by 20 min UV exposure. Isopropyl alcohol washing was performed again to remove the remaining ink. The mold was then plasma-treated for 2 min to activate the surface followed by functionalization with trichloro(1*H*,1*H*,2*H*,2*H*-perfluorooctyl) silane (Sigma-Aldrich, 448931) in a vacuum chamber for 15 min to minimize stiction during subsequent molding steps.

#### 2.5.2. Concaved Channel Molding

For the concaved channel fabrication, Polydimethylsiloxane (PDMS, Dow Corning Sylgard 184) and its crosslinker were mixed at the ratio of 10 to 1. The mixture was then poured on the negative mold and degassed in a vacuum chamber for 30 min and cured in 70 °C for 5 h. Upon being cured, the PDMS substrate was peeled off from the negative mold and wash in 70% EtOH for subsequent use. To coat the PDMS substrate with collagen to facilitate cell-substrate adhesion, PureCol^®^ EZ Gel (Advanced BioMatrix, San Diego, CA, USA, 5074-G) was diluted to the final concentration of 0.5 mg/mL and then added to the surface of the PDMS substrate. The collagen-treated PDMS substrate was then incubated at 37 °C for an hour to allow sufficient adsorption. The PDMS substrate was washed by PBS afterward three times to remove the unadsorbed collagen. Collagen coating was performed on the same day before cell seeding.

### 2.6. Imaging Analysis

Image analyses were performed using Fiji/ImageJ software [16]. For cortical actin analysis, the Z-stack images were first projected to the single x-y plane with maximum intensity. Cell edges were determined using the fluorescence which originated from cortical F-actin and marked by hand-tracing. The fluorescence intensity of pixels located on the cell edge was summed and divided over the number of total pixels consisting of the cell edge, to obtain the average cortical F-actin intensity. The pixels of the cell excluding the cell edge were marked and the fluorescence intensity of these pixels was summed and divided over the total number of pixels to obtain the average F-actin intensity in the rest of the cell. To obtain the relative cortical actin density, the average intensity of cortical F-actin along the cell edge was divided by the average F-actin intensity in the rest of the cell. For the F-actin distribution along the line, the straight lines were drawn to cross cells. Then, the Plot Profile function was used to get the intensity along the line. To determine the relative abundance of E-cadherin and the degree of myosin phosphorylation along the cell edge, the z-stack images were first projected to the single x-y plane with maximum intensity. Cell edges were determined using the fluorescence which originated from E-cadherin or pMLC respectively and marked by hand-tracing. The fluorescence intensity of pixels located on the cell edge was summed and divided over the number of total pixels consisting of the cell edge, to obtain the average E-cadherin or pMLC intensity. For the total MLC analysis, cell edges were traced by hand. The fluorescence intensity of pixels located within the cell edge was summed and divided over the number of total pixels within the cell edge, to obtain the average total MLC intensity. For curvature verification, the x-z sections of z stacks were obtained from using Orthogonal Views function in Fiji. The Multi-point tool was used to trace along the curved surface from the fluorescence which originated from F-actin. The Fit Circle function in Fiji was then used to fit the traced border of the curved channel with the minimal circle. The area of the fitted circle was measured, and the radius of the circles was then calculated from the obtained area.

### 2.7. Statistical Analysis

The bar graphs were shown as the mean ± standard deviation (SD). Whether significant differences exist between different treatments was determined using Student’s t-test: * indicates *p* < 0.05; ** indicate *p* < 0.01; *** indicates *p* < 0.001.

## 3. Results and Discussion

### 3.1. Epithelial Cells Formed Continuous Epithelium on Curved Surfaces

**Figure 1:**
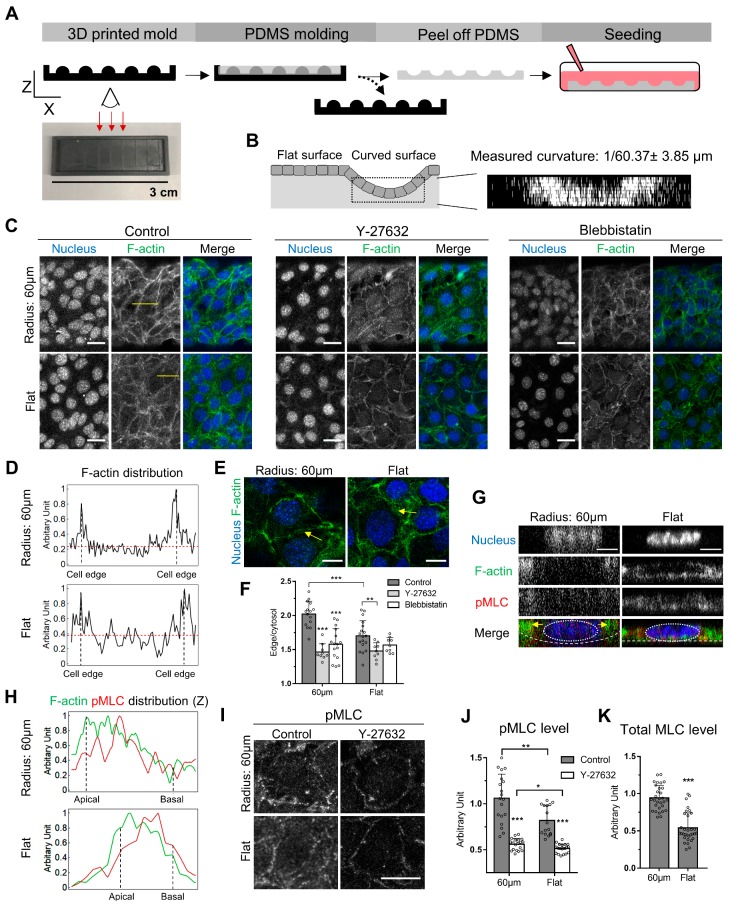
Curvature-dependent cortical actin is regulated by myosin phosphorylation in EpH4-EV cells. (**A**) The fabrication steps of the channels with concaved curvature of 1/60 µm using 3D printed molds are shown. (**B**) The schematic shows how the continuous epithelium is formed over the substrate on both the curved and flat surfaces. The reconstructed XZ images were used to confirm that the desired curvature was achieved (n = 6). (**C**) Different F-actin distribution patterns between cells on the curved and flat surfaces were distinguished by fluorescent phalloidin staining. The cells on the curved surface and the flat surface were treated with DMSO, Y-27362, or Blebbistatin for 2 h prior to the staining of F-actin. The nuclei were marked by DAPI staining. (**D**) The line scans of fluorescent F-actin intensity in selected cells on the curved and flat surfaces are compared. The intensity profile was extracted along the yellow lines indicated in (**C**). The F-actin intensity was normalized by dividing the fluorescent intensity of every pixel in the line scan over the intensity of the brightest pixel. The red dotted lines mark the average intensity of the cytoplasmic F-actin, excluding the cell edge. (**E**) Comparable levels of F-actin intensity at the lateral side of the cell–cell contacts were detected. The contrast of the images was adjusted here differently than (**C**), for the purpose of visualization. (**F**) The intensity ratio between cortical and cytoplasmic F-actin was quantified in cells treated with DMSO, Y-27632, and Blebbistatin. For cells on the curved surfaces, n = 15, 9, 16; for cells on the flat surface n = 18, 9, 9, respectively. (**G**) The x-z view of a cell-cultured on the curved surface and another cell on the flat surface with pMLC and F-actin staining. (**H**) The line scans of fluorescent F-actin (green) and pMLC (red) intensity in cells shown in (**G**) on the curved and flat surfaces are compared. The lines were drawn perpendicularly at the right side of the cells. (**I**) pMLC was stained in cells on the curved and flat surfaces with or without Y-27632 treatment. (**J**) The average pMLC intensity was determined in cells treated with DMSO and Y-27632. For cells on the curved surface, n = 19, 19; for cells on the flat surface, n = 16, 21, respectively. (**K**) The average total MLC intensity was determined in cells cultured on the curved and flat surface. For cells on the curved surface, n = 30; for cells on the flat surface, n = 32. Scale bars: (**C**,**I**) 25 µm, (**E**) 10 µm, (**G**) 5 µm. Error bars represent SD. Whether significant differences exist between different treatments was determined using Student’s t-test: * indicates *p* < 0.05; ** indicate *p* < 0.01; *** indicates *p* < 0.001.

To evaluate the effects of curvature at the cellular level, mouse mammary gland epithelial cells (EpH4-EV) were cultured on open channels with the curvature of 1/60 µm, in mimicry of the curved surface belonging to the ductal structure of the mammary gland [17,18]. The PDMS-based open channels were fabricated using a 3D-printed mold (Figure 1A). By 3D image reconstruction, we observed that the average curvature of the open channels reached 1/60 ± 3.85 μm (Figure 1B), indicating our fabrication method is reliable with small deviations. To control for the effect of substrate elasticity, the epithelial cells on the flat intervaled region between the concaved channels were used as control for comparison.

### 3.2. Curvature-Dependent Cortical Actin Increase Is Regulated by Myosin II Phosphorylation

We first examined whether cells grown on the curved surface exhibit different phenotypes compared to cells grown on the flat surface. Cells were stained with fluorescently labeled phalloidin to visualize the F-actin. Our rationale is that since actin cytoskeleton determines the cell shape conforming to the geometry of the substrate, the organization and the distribution of the cells might reflect the effect imposed by the substrate curvature. By comparing EpH4-EV cells growing on the curved surface and flat substrate, a higher intensity of cortical F-actin, positioned along the cell edge, was observed compared to that of cells cultured on the flat surface (Figure 1C). The ratio of cortical F-actin intensity was obtained by dividing the average fluorescence intensity of the cortical F-actin by that detected in the rest of the cell. We found that cells on the curved surface exhibit a 1.2-fold higher cortical actin density compared to the flat surface (Figure 1D,F). Interestingly, a comparable increment in cortical actin density has been observed in ovarian cancer cells with high invasiveness when compared to the control [19], suggesting cells are even sensitive to small changes in cortical actin density and manifest such sensitivity in downstream phenotypical traits such as motility. Cortical actin assembly is regulated by myosin II phosphorylation. The phosphorylated myosin II exerted the contractile force on the actin cortex to regulate its membrane rigidity and plasticity [20]. Therefore, we examined whether myosin II contributes to higher cortical actin formation in cells on the curved surface by treating cells with Y-27632 or Blebbistatin. Y-27632 is a Rho-associated coiled-coil containing protein kinase (ROCK) inhibitor [21], which results in the downstream inhibition of myosin II phosphorylation. Blebbistatin is a myosin II inhibitor [22]. ROCK is an upstream regulator of myosin II, and inhibition of ROCK results in inhibition of myosin II phosphorylation [23]. We found that Y-27632 and Blebbistatin, both resulting in myosin inhibition, reduced the cortical actin intensity comparable to the level of control cells on the flat substrate (Figure 1C,F). It has been shown that higher myosin activity is required to maintain the cortical stability in regions which experience higher surface tension in the cell [24]. Because the normal surface tension is higher at the curved surface [25], we examined whether myosin II activity is accordingly elevated by immunofluorescence using anti-pMLC antibody to visualize the extent of myosin II phosphorylation. A higher level of myosin II phosphorylation was detected in cells on the curved surface compared to the flat substrate (Figure 1I,J). It was also observed that Y-27632 treatment abolished the elevated myosin II phosphorylation on the curved surface (Figure 1I,J), as well as the increased cortical actin density, further implying actomyosin activity is required for the curvature sensing machinery at the scale of tens of microns (i.e., at the multicellular level). Notably, it has been shown that Rho activation promotes stress fiber formation and cell–cell alignment in fibroblast on the convexed surface [14], suggesting Rho activation might be involved in curvature sensing at the multicellular level. It should be noted while myosin activity is a valid indicator of actomyosin forces generated by the cells [24,26,27,28,29,30], traction force measurement techniques suitable for observations on curved substrates will be desirable and should be developed to directly measure the forces.

To examine whether cells adapt to the curved surface by regulating the expression of myosin II, in addition to modulating its activity, we performed the immunofluorescence targeting MLC (Figure 1K). We found that MLC expression in cells on the curved surface was two-fold higher than that in cells on the flat surface. This result suggests cells respond to the curvature by both increasing the gene expression and the activity of myosin II. Interestingly, the fold-increase in terms of myosin expression is higher than the fold-increase of myosin phosphorylation, indicating myosin light chain kinase (MLCK) activity is not modulated by the curvature to maintain a constant ratio between phosphorylated and total myosin.

When epithelial cells migrate collectively with a concaved leading front at the x-y plane, the actomyosin network is enriched at the leading edge of the cells in the front row [31], resulting in net forces driving cells moving forward. In the case of immotile cells adhered to, the curved surface force balance needs to be achieved [32], so that the integrity of cell–cell adhesion can be sustained. We have designed the culturing condition so that the epithelium on the curved surface was in a jammed steady state and did not migrate upwards to form finger-like protrusions [33]. Therefore, we postulated the force balance should be achieved between actomyosin contractility and the normal stress exerted by the curved surface. At the lateral side of the cell–cell contact, F-actin in the cell on the curved surface was at a comparable level to that on the flat surface (Figure 1E,H). We observed that F-actin and phosphorylated myosin were more enriched closer to the apical side when cells are cultured on the curved surface, whereas no enrichment was observed on the flat surface (Figure 1G,H), indicating that such polarized distribution facilitates the balance between normal stress and the contractility of the actomyosin network.

### 3.3. Cell-Cell Adhesion Is Required in the Curvature-Dependent Regulation of Cortical Actin Assembly

**Figure 2:**
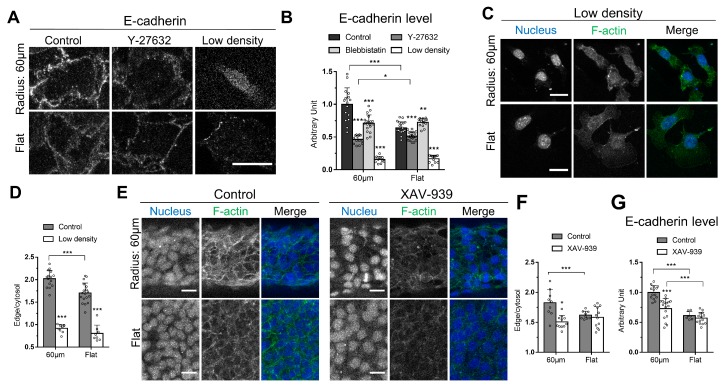
Cell–cell adhesion is involved in the elevated cortical actin assembly on the curved surface. (**A**) Cells treated with DMSO or Y-27632 on the curved and flat surfaces were stained with an anti-E-cadherin antibody. Cells with lower initial seeding density (control: 5 × 10^5^, low density: 10^4^) were also examined. (**B**) The average E-cadherin intensity levels were measured for the DMSO-treated, Y-27632-treated, Blebbistatin-treated, and lower seeding density group. For cells on the curved surfaces, n = 16, 14, 19, 12; for cells on the flat surfaces, n = 20, 21, 15, 12, respectively. (**C**) The images show the F-actin distribution of cells on the curved and flat surfaces in the lower seeding cell density. (**D**) The intensity ratio between cortical and cytoplasmic F-actin was quantified in cells with different initial seeding densities. For cells on the curved surfaces, n = 15, 9; for cells on the flat surfaces, n = 18, 8, respectively. (**E**) The cells on the curved surface and flat surfaces were treated with DMSO or XAV-939 for β catenin inhibition and were followed by phalloidin and DAPI staining. (**F**) The intensity ratio between cortical and cytoplasmic F-actin was quantified in cells treated with DMSO and XAV-939. For cells on the curved surfaces, n = 9, 11; for cells on the flat surfaces, n = 9, 12, respectively. (**G**) The average E-cadherin intensity levels were measured for the DMSO-treated and XAV-939-treated group. For cells on the curved surfaces, n = 14, 17; for cells on the flat surfaces, n = 7, 14, respectively. Scale bars: 25 µm. Error bars represent SD. Whether significant differences exist between different treatments was determined using Student’s t-test: * indicates *p* < 0.05; ** indicate *p* < 0.01; *** indicates *p* < 0.001.

We then examined whether E-cadherin, another protein known to localize at the cell edge, also exhibits a similar curvature-dependent pattern. E-cadherin usually locates at the adherens junction, where it forms the E-cadherin-catenin complex to connect F-actin to the adherens junction at the interface of cell–cell adhesion. Cells were stained with anti-E-cadherin antibody to visualize the adherens junction. The structural integrity of the epithelium is critically maintained by the adherens-cytoskeleton networks spanning the cell population comprising the epithelium [34]; 1.6-fold higher E-cadherin was detected in cells on the curved surface, compared to their counterparts growing on the flat surface (Figure 2A,B). Interestingly, Y-27632 and Blebbistatin treatments reduced E-cadherin expression in cells on the curve surface to the level observed in the cells on the flat surface (Figure 2A,B), indicating that both myosin II and E-cadherin are associated with the cortical actin network assembly, and signaling pathways governing myosin activity might contribute to the recruitment of E-cadherin at the adherens junction by a feedback loop.

The correlation between E-cadherin expression and cortical actin density prompted the question whether the state of cell–cell adhesion is associated with cortical actin density regulation in a curvature-dependent manner. To answer this question, cells were seeded on to the curved surface at lower density, 10^4^ cells in total, so that a continuous epithelium would not form 1 day after seeding, as opposed to the case when higher seeding density was used. Very little cortical actin staining by fluorescent phalloidin could be detected in the cells on the curved surface. The result was similar in the cells on the flat surface (Figure 2C,D). Furthermore, in the lower density group, the reduction of E-cadherin expression was observed on both the curved and flat surface (Figure 2A,B). The result demonstrates the importance of cell–cell adhesion in curvature-dependent cortical actin assembly, and the important role of cell–cell adhesion for the cell to respond to curvature.

Prominent junctional localization of E-cadherin is observed when tension between cells is high [35]. We saw higher junctional localization in the cell on the curved surface, which is likely caused by high tension between cells when on the curved surface. Higher tension between cells requires increased cell–ECM adhesion to reach the force balance [36,37,38]. Based on these observations, it was deduced that if the cell–ECM adhesion is not reinforced in proportion to the increased tension between cells on the curved surface, the epithelium might adjust its height and spread area to achieve balance [36], or detach from the substrate. Interestingly, reduced focal adhesions were observed when cells were cultured on the curved surface compared to cells on the flat control [15]. In addition, with lower cell–ECM adhesion and higher cell–cell tension in cells on the curved surface, detachment of Madin-Darby Canine Kidney cells has been observed 24–48 h after seeding [12]. Our observation is in agreement with this principle, though it should be noted that the detachment was not observed in our study, probably because the epithelium was cultured and fixed within 24 h after seeding. The difference of cell–matrix interactions due to curvature change, despite its subtlety at the single cellular level, can be integrated through E-cadherin signaling and result in a prominent phenotype at the multi-cellular level.

### 3.4. β-Catenin Is Involved in the Curvature-Dependent Regulation of Cortical Actin Assembly

Next, we examined the involvement of E-cadherin-mediated signaling pathway in regulating cortical actin assembly on the curved surface. Cells were treated with the drug XAV-939, a β-catenin inhibitor [39]. XAV-939 had been used to specifically target β-catenin signaling [40,41,42,43,44,45,46,47,48] because it stabilizes Axin, the destruction complex targeting β-catenin for ubiquitylation and the subsequent proteasomal degradation, by inhibiting poly(ADP)-ribosylating enzymes tankyrase 1 and tankyrase 2 [39]. β-catenin binds tightly to the cytoplasmic domain of E-cadherin [49], then the E-cadherin/β-catenin complex binds to α-catenin, which in turn is connected to actin cytoskeleton [50]. XAV-939-treated cells on the curved surface showed significant reduction in cortical actin density (Figure 2E,F). The reduced cortical actin intensity was as low as the treated, or untreated cells on the flat surface, suggesting β-catenin does regulate the cortical actin assembly in cells on the curved surface. β-catenin has been considered dispensable in the context of forming adherens junction, given that silencing β-catenin does not affect cell–cell adhesion. It is because α- and β-catenin are redundant in this regard [51,52]. Here, we found that β-catenin inhibition does result in a detectable response to curvature-dependent cortical actin assembly, implying α-catenin cannot compensate for the function of β-catenin in the context of curvature sensing. Furthermore, the E-cadherin expression level was decreased by approximately 25% in cells treated with XAV-939 (Figure 2G), suggesting a potential feedback regulation existing between β-catenin and E-cadherin, which cannot be completely replaced by α-catenin. Our result agrees with the observation that interaction between β-catenin, but not α-catenin, and vinculin is essential for regulating the distribution of E-cadherin at the adherens junction [53].

In summary, we observed that cells in epithelium collectively respond to the curvature of similar dimensions as the mouse mammary ducts by comparing the observations in cells cultured on the flat and concaved surface with 60-µm radius. In particular, we found that actomyosin cytoskeleton and adherens junctions are affected by the curvature. Of note, to comprehensively understand the effect of the curvature on phenotypes of the cells, substrates of different curvatures should be tested so that a quantitative relationship between the curvature and the phenotype can be established.

**Figure 3:**
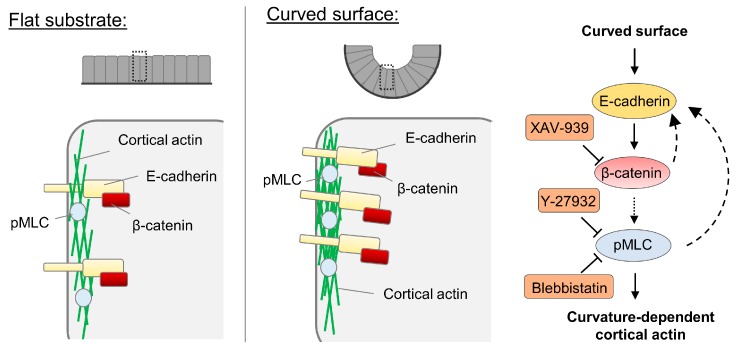
Proposed model for the increased cortical actin assembly in epithelium on the curved surface. The capacity of epithelial cells collectively sensing the curvature at the length scale of tens of micros is manifested by elevated E-cadherin and myosin phosphorylation along the cell edge. Our results indicate that through β-catenin-dependent signaling, cortical actin assembly is increased. Feedback regulation of E-cadherin by the myosin activity and β-catenin is also proposed here based on the results.

The cortical actin density is increased in cells on the curved surface via the regulation involving myosin II and E-cadherin-mediated signaling (Figure 3). It should be noted that though various molecules have been previously reported to be curvature-sensitive, the curvatures used in those previous studies are of the length scale of sub-microns [8]. Our result demonstrates that for curvatures at the multicellular level length scale, adherens junctions and cortical actin density are the cellular components potentially responsible for curvature sensing and the corresponding downstream adaptations. Similarly, adherens junctions also were observed to be involved in the collective durotaxis, which is an emerging behavior at the multicellular level [54]. In other words, the substrate rigidity gradient sufficient to induce collective durotaxis is not sufficient for single cells to sense and migrate in a durotactic fashion. However, epithelium as a whole can sense it through E-cadherin.

In this study, we observed that curvatures govern the cytoskeletal organization, which is important in many aspects of tumor progression. In agreement of our interpretation, cancer cell migration or cancer stem cell formation were observed to be dependent on the mechanical stress as a function of local curvatures in ductal structures [2,3]. We used normal mammary gland epithelial cells to study the effects of the curvature whose value is relevant to the ductal geometry. The curvature effect on breast cancer cells might result in different cell behaviors, where E-cadherin and α-catenin expression level is already low [55]. Further investigation is required to elucidate the exact molecular mechanism underlying these curvature-dependent behaviors in both physiological and pathological conditions.
